# Clinical Accuracy and Safety Concerns Following GPT-5 Public Demonstration in Cancer Care

**DOI:** 10.1007/s10916-025-02312-x

**Published:** 2025-11-29

**Authors:** Ivan Capobianco, Andrea Della Penna, André L. Mihaljevic, Michael Bitzer, Carsten Eickhoff, Derna Stifini

**Affiliations:** 1 Department of General, Visceral and Transplant Surgery, University Hospital Tuebingen, Tuebingen, Germany; 2 Department of Internal Medicine I, University Hospital Tuebingen, Tuebingen, Germany; 3 Center for Digital Health, University Hospital Tuebingen, Tuebingen, Germany

## Abstract

OpenAI’s GPT-5 demonstration showed a patient uploading pathology reports to guide treatment decisions, though privacy implications were not addressed. We evaluated GPT-5 against 100 gastrointestinal oncology cases with tumor-board validation and found identical 85% concordance to GPT-4o, contradicting superiority claims. We recommend mandatory accuracy disclosures and regulatory oversight for AI health demonstrations to protect patient safety and privacy.

## Main Text

### Brief-Lede

Large language models, or LLMs, have become increasingly popular tools in medicine over the past two years. They can provide clinical support, help patients learn about their diseases, and even assist in making treatment decisions. Healthcare applications of AI have expanded rapidly, with cancer care emerging as a particularly active area of development due to the complexity of treatment decisions and the need for personalized approaches.

In August 2025, OpenAI introduced GPT-5, positioning it as a significant advancement in AI capabilities for healthcare applications. They called it their best model for health-related questions, claiming superior performance in medical reasoning and clinical decision support. The launch strategy was notable for its emphasis on real-world healthcare applications rather than general AI capabilities.

The launch featured a compelling patient case: a cancer patient who uploaded her pathology report to ChatGPT to seek information about her diagnosis and subsequently used the AI’s guidance to resolve disagreement between her physicians on radiation therapy. OpenAI framed GPT-5 as a “thought partner” that could synthesise context, proactively raise missing issues, and guide patients in decision-making. This marketing approach represented a shift toward positioning AI as an active participant in clinical decision-making rather than merely an informational tool.

The story was powerful and hopeful. It showed AI empowering patients to take charge of their health and potentially democratizing access to complex medical expertise. The narrative resonated with broader themes of patient autonomy and shared decision-making that have become central to modern healthcare. While compelling as a narrative, this raises serious questions about privacy, patient safety, and the accuracy of such tools when applied to high-stakes clinical decisions.

### Evidence-Based Arguments

#### Performance Evaluation of GPT-5 Versus Previous Models

To independently assess GPT-5’s claimed superiority, we tested it alongside GPT-4o-mini and GPT-4o in a single-center, rapid audit study using a dataset of 100 gastrointestinal oncology cases, each with tumour-board-validated recommendations. Cases were retrospectively selected from our single-center gastrointestinal tumor board following ethics approval (No. 273/2024BO1), excluding patients enrolled in clinical trials or with atypical diagnoses, and using simple random sampling to select 20 patients per tumor subgroup. Overall inclusion dates ranged from March 2022, to December 2023. The dataset included equal representation of oesophageal, pancreatic, gastric, colorectal, and hepatobiliary cancers, with a median patient age of 64.5 years (IQR 56–71) and a male-to-female ratio of 64:36.

All models saw the identical anonymised clinical case narrative. Prompts were structured to assign the role of a multidisciplinary gastrointestinal oncology tumor board and to request a concise, guideline-style therapeutic recommendation (in German) in one to two sentences. Inference was performed via the OpenAI API in August 2025. For GPT-4o and GPT-4o-mini, generation parameters were explicitly set to temperature = 0.8 and Top *P* = 1, chosen to approximate the behaviour of the ChatGPT web interface. GPT-5 operates with a fixed internal sampling temperature, which is not user-adjustable. Model identifiers were: “GPT-5”, “GPT-4o”, and “GPT-4o-mini”.

Both tumour board and GPT recommendations were categorized into nine therapeutic classes (e.g., supportive care, surgery, systemic therapy), with classifications performed independently by two blinded clinicians resolving discrepancies by consensus. Concordance was defined as matching the same therapeutic category. When the tumor-board provided multiple options, GPT recommendations were considered concordant if at least one matched.

**Fig. 1:**
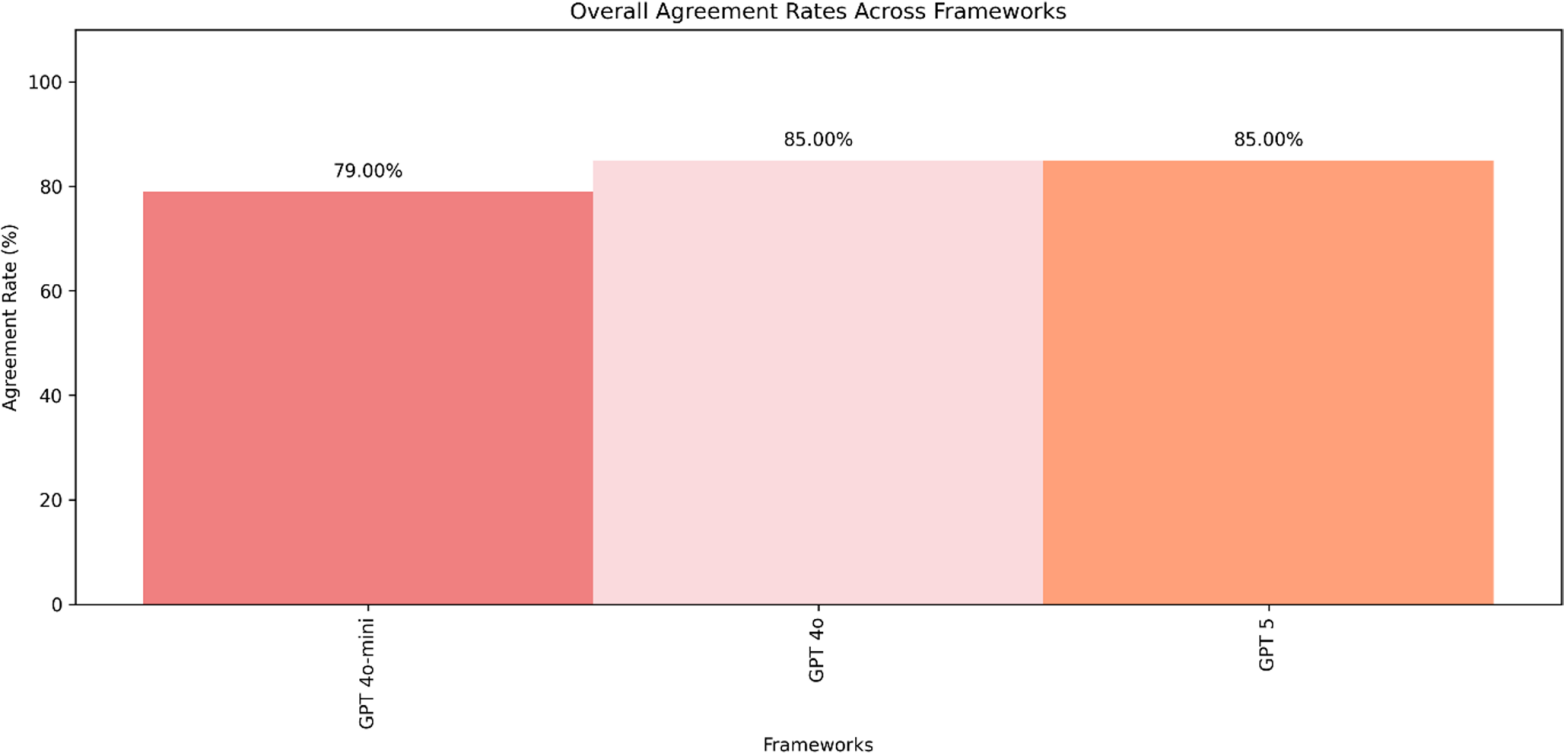
Overall concordance between GPT-4o-mini, GPT-4o, and GPT-5 versus tumour board recommendations

**Fig. 2:**
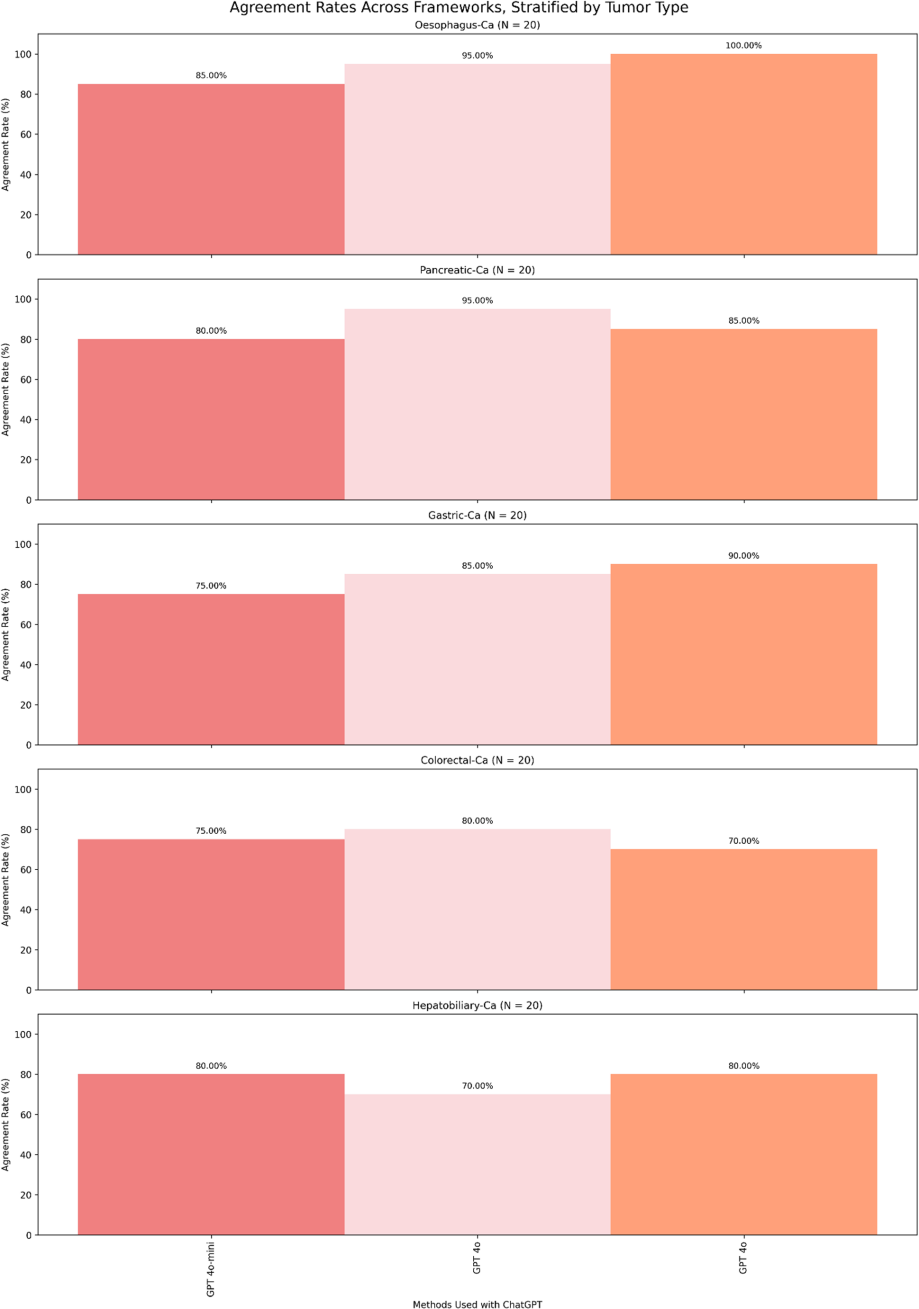
Considerable variability can be observed in performance between different models and tumor types, with concordance ranging from 75–85% for GPT-4o-mini, 80–95% for GPT-4o, and 70–100% for GPT-5 across the different tumor types, with exact numerators, denominators, and 95% confidence intervals for each tumor type as follows: •Oesophageal: GPT-4o-mini 85.0% (17/20, 95% CI 63.96–94.76%), GPT-4o 95.0%(19/20, 95% CI 76.39–99.11%), GPT-5 100.0% (20/20, 95% CI 83.89–100.00%); •GPT-4o-mini 80.0% (16/20, 95% CI 58.40–91.93%), GPT-4o 95.0% (19/20, 95% CI76.39–99.11%), GPT-5 85.0% (17/20, 95% CI 63.96–94.76%); •Gastric: GPT-4o-mini 75.0% (15/20, 95% CI 53.13–88.81%), GPT-4o 85.0% (17/20,95% CI 63.96–94.76%), GPT-5 90.0% (18/20, 95% CI 69.90–97.21%); •Colorectal: GPT-4o-mini 75.0% (15/20, 95% CI 53.13–88.81%), GPT-4o 80.0%(16/20, 95% CI 58.40–91.93%), GPT-5 70.0% (14/20, 95% CI 48.10–85.45%); •Hepatobiliary: GPT-4o-mini 80.0% (16/20, 95% CI 58.40–91.93%), GPT-4o 70.0%(14/20, 95% CI 48.10–85.45%), GPT-5 80.0% (16/20, 95% CI 58.40–91

Overall concordance with tumour board recommendations was 79/100 for GPT-4o-mini (95% CI 70.02–85.83%), and 85/100 for both GPT-4o and GPT-5 (95% CI 76.72–90.69% for each; see Fig. 1). Figure 2 shows model performance by tumor type with exact numerators, denominators, and 95% confidence intervals, highlighting heterogeneity across cancers. While GPT-5 demonstrated domain-specific gains, its overall accuracy was identical to GPT-4o and showed weaknesses in colorectal cancer—contradicting the claim of consistent superiority. Cochran’s Q test and pairwise McNemar tests revealed no significant differences between the three models (*p* ≥ 0.21 for all comparisons). Repeated runs under the same conditions of each model on a balanced 20-case subset didn’t change the result, with maximum variability of 2 cases and no change in relative performance.

Published studies confirm that LLMs can match or even beat humans in some narrow oncology tasks. For example, extracting data from radiology reports with over 95% accuracy (1, 2). However, for clinical decision-making, even the best models report error rates above 15% (3–5), with risks of outdated, fabricated, or hallucinated content (6–8). These limitations become particularly problematic in oncology, where treatment decisions often involve complex risk-benefit calculations and consideration of patient-specific factors that may not be fully captured in clinical documentation.

To put this into perspective, an 85% concordance rate, as we found, means that roughly 1 in 6 recommendations could be incorrect. In oncology practice, where treatment decisions can dramatically impact survival outcomes, quality of life, and healthcare costs, this error rate raises significant concerns about patient safety when AI tools are used without clinical supervision.

These accuracy limitations become particularly concerning when combined with the privacy risks demonstrated in the GPT-5 launch.

## Privacy and Data Protection Risks

The case shown in the GPT-5 launch event normalizes uploading identifiable medical documents to a commercial LLM platform without discussing these risks, implicitly endorsing behaviours that could compromise confidentiality.

Such actions introduce multiple vulnerabilities:

- Data transfer outside secure clinical environments, potentially breaching health privacy laws (e.g., GDPR in Europe, HIPAA in the US).
- Opaque storage and processing—patients may not be fully aware of where and how their data is used.
- Potential model training exposure, if user-uploaded data is retained.

In the US context specifically, uploads to non-HIPAA-covered commercial AI services are not protected by HIPAA at all, creating an even larger regulatory vacuum than commonly perceived.

Furthermore, medical reports contain numerous identifiable elements beyond patient names, including physician identifiers, institutional headers, specific dates, and unique case numbers that could enable re-identification even when names are removed. The cross-border nature of cloud computing adds additional complexity, as data uploaded to US-based platforms may be subject to different privacy protections than those guaranteed under European or other national frameworks.

The convergence of factors— (1) unsupervised clinical decision-making, (2) privacy risks from document upload, and (3) overstated performance claims—highlights the need for stronger governance:

- Mandatory transparency in AI health product presentations, including limitations, error rates, and scope of validation.
- Prohibition of patient-identifiable uploads to non-health-regulated AI platforms, unless explicit, informed consent and secure processing guarantees are in place.
- Independent benchmarking of health claims using standardised, publicly available datasets across cancer types and decision categories.

## Clinical Implementation Risks

While the portrayal of AI in the GPT-5 demonstration underscores empowerment and accessibility, it also suggests that patients might bypass established clinical safeguards, replacing evidence-based multidisciplinary consensus with AI-mediated reasoning. Multidisciplinary teams of experts exist specifically to reduce errors and cognitive biases that can affect individual clinical judgment. These teams integrate expertise from medical oncology, surgical oncology, radiation oncology, pathology, radiology, and other specialties to ensure comprehensive treatment planning.

Replacing this collaborative consensus with AI advice, especially without clinical supervision, introduces systemic risks that extend beyond individual cases. This demonstration implicitly endorses AI as a primary decision-making tool, contradicting established clinical care standards that emphasize multidisciplinary review and shared decision-making between patients and their care teams.

Promoting AI as a standalone decision-maker in complex cancer care is premature and poses documented patient safety risks. Current AI models lack the ability to perform physical examinations, assess functional status, understand psychosocial factors, or integrate real-time clinical observations that are crucial for optimal treatment planning. They also cannot account for patient preferences, values, and goals of care that require nuanced human interaction.

Moreover, models tend to express outputs with high confidence, regardless of correctness, which can mislead patients and non-expert users. This confidence bias can be particularly dangerous in oncology, where patients facing life-threatening diagnoses may be especially vulnerable to authoritative-sounding advice. If a patient’s case falls among those incorrect suggestions, it could lead to inappropriate treatment decisions, undermine trust between patient and clinician, and cause significant harm—including delays in optimal care, exposure to unnecessary toxicity, increased healthcare costs, and emotional distress for patients and their families.

When such tools are publicly demonstrated without explicit caveats about limitations and appropriate use, patients may interpret them as clinically reliable substitutes for professional medical advice. This undermines the carefully developed multidisciplinary care models that exist precisely to mitigate cognitive biases, incomplete information, and the inherent uncertainty in cancer treatment decisions.

## Forward-Looking Recommendations

We acknowledge the clear benefits of developing faster, more accessible tools to support patient care and the promising role of large language models in healthcare transformation. AI technologies have demonstrated value in medical education, clinical documentation, and patient engagement. However, these tools must be reliable and meet rigorous clinical standards before being promoted for direct patient use in high-stakes decisions. Accuracy and safety cannot be compromised in the pursuit of technological advancement.

The GPT-5 launch highlights both the exciting potential and the serious risks of generative AI in oncology practice. AI can help patients better understand their condition, navigate complex medical information, and feel more empowered in their healthcare journey. These benefits should not be dismissed or minimized. However, promoting unsupervised use for treatment decisions, especially when privacy risks exist and validation remains incomplete, puts patient safety at risk and may ultimately undermine trust in both AI tools and healthcare providers.

Our independent evaluation found that, despite marketing claims and some domain-specific improvements, GPT-5 did not perform better overall than GPT-4o in matching tumour board decisions across gastrointestinal cancers. The identical 85% concordance rates suggest that claims of superior clinical reasoning may be overstated, at least in the oncology domain we tested.

Given the current state of technology and our findings, it is crucial that clinicians remain at the center of patient care while AI tools serve as supportive rather than directive instruments. Proper training and clear clinical guidelines are essential to ensure healthcare professionals understand both the strengths and limitations of AI tools, can accurately interpret relevant data and literature, and use these technologies responsibly within established multidisciplinary teams to effectively discuss treatment options with patients.

Future research should focus on developing robust validation frameworks for AI health tools, establishing clear regulatory pathways for clinical AI applications, and creating evidence-based guidelines for appropriate AI use in oncology practice. Only through such systematic approaches can we harness the benefits of AI while protecting patient safety and maintaining the trust that is fundamental to effective healthcare delivery.

## Data Availability

The data that support the findings of this study are not publicly available due to reasons of sensitivity and are available from the corresponding author upon reasonable request. Data are located in controlled access data storage at University Hospital Tübingen.

## References

[1] Fink MA, Bischoff A, Fink CA, Moll M, Kroschke J, Dulz L, et al. Potential of ChatGPT and GPT-4 for Data Mining of Free-Text CT Reports on Lung Cancer. Radiology. 2023;308(3):e231362. 37724963 10.1148/radiol.231362

[2] Chen D, Alnassar SA, Avison KE, Huang RS, Raman S. Large Language Model Applications for Health Information Extraction in Oncology: Scoping Review. JMIR Cancer. 2025;11:e65984. 40153782 10.2196/65984 PMC11970800

[3] Benary M, Wang XD, Schmidt M, Soll D, Hilfenhaus G, Nassir M, et al. Leveraging Large Language Models for Decision Support in Personalized Oncology. JAMA Netw Open. 2023;6(11):e2343689. 37976064 10.1001/jamanetworkopen.2023.43689 PMC10656647

[4] Lammert J, Dreyer T, Mathes S, Kuligin L, Borm KJ, Schatz UA, et al. Expert-Guided Large Language Models for Clinical Decision Support in Precision Oncology. JCO Precis Oncol. 2024;8:e2400478. 39475661 10.1200/PO-24-00478

[5] Rydzewski NR, Dinakaran D, Zhao SG, Ruppin E, Turkbey B, Citrin DE, et al. Comparative Evaluation of LLMs in Clinical Oncology. NEJM AI. 2024;1(5). 10.1056/aioa2300151 PMC11315428 39131700

[6] Chen D, Avison K, Alnassar S, Huang RS, Raman S. Medical accuracy of artificial intelligence chatbots in oncology: a scoping review. Oncologist. 2025;30(4). 10.1093/oncolo/oyaf038 PMC12032582 40285677

[7] Berman E, Sundberg Malek H, Bitzer M, Malek N, Eickhoff C. Retrieval Augmented Therapy Suggestion for Molecular Tumor Boards: Algorithmic Development and Validation Study. J Med Internet Res. 2025;27:e64364. 40053768 10.2196/64364 PMC11923455

[8] Yalamanchili A, Sengupta B, Song J, Lim S, Thomas TO, Mittal BB et al (2024) Quality of Large Language Model Responses to Radiation Oncology Patient Care Questions. JAMA Netw Open. 7(4):e244630 38564215 10.1001/jamanetworkopen.2024.4630 PMC10988356

